# Dissipation and Safety Analysis of Dimethomorph Application in Lychee by High-Performance Liquid Chromatography–Tandem Mass Spectrometry with QuEChERS

**DOI:** 10.3390/molecules29081860

**Published:** 2024-04-19

**Authors:** Siwei Wang, Xiaonan Wang, Yanping Liu, Qiang He, Hai Tian

**Affiliations:** 1 Plant Protection Research Institute, Guangdong Academy of Agricultural Sciences, Key Laboratory of Green Prevention and Control on Fruits and Vegetables in South China, Ministry of Agriculture and Rural Affairs, Guangdong Provincial Key Laboratory of High Technology for Plant Protection, No. 7 Jinying Road Tianhe District, Guangzhou 510640, China; wangsiwei@gdaas.cn (S.W.); wangxiaonan@gdaas.cn (X.W.); 2 Guangdong Quality Safety Center of Agricultural Products (Guangdong Green Food Development Center), Department of Agriculture and Rural Affairs of Guangzhou, No. 135 Xianlie East Road Tianhe District, Guangzhou 510500, China; 3 Analysis and Testing Center, Key Laboratory of Quality and Safety Control of Subtropical Fruits and Vegetables, Ministry of Agriculture and Rural Affairs, Chinese Academy of Tropical Agricultural Science, No. 4 Xueyuan Road, Longhua District, Haikou 571101, China

**Keywords:** dimethomorph, lychee, terminal residues, dissipation dynamic, risk quotient

## Abstract

This study presents a method for analyzing dimethomorph residues in lychee using QuEChERS extraction and HPLC-MS/MS. The validation parameters for this method, which include accuracy, precision, linearity, and recovery, indicate that it meets standard validation requirements. Following first-order kinetics, the dissipation dynamic of dimethomorph in lychee was determined to range from 6.4 to 9.2 days. Analysis of terminal residues revealed that residues in whole lychee were substantially greater than those in the pulp, indicating that dimethomorph residues are predominantly concentrated in the peel. When applied twice and thrice at two dosage levels with pre-harvest intervals (PHIs) of 5, 7, and 10 days, the terminal residues in whole lychee ranged from 0.092 to 1.99 mg/kg. The terminal residues of the pulp ranged from 0.01 to 0.18 mg/kg, with the residue ratio of whole lychee to pulp consistently exceeding one. The risk quotient (RQ) for dimethomorph, even at the recommended dosage, was less than one, indicating that the potential for damage was negligible. This study contributes to the establishment of maximum residue limits (MRLs) in China by providing essential information on the safe application of dimethomorph in lychee orchards.

## 1. Introduction

Lychee (*Litchi chinensis* Sonn.) is a southern specialty fruit from China and has been grown there for over 2000 years. China has the most significant number of lychee plants and produces the highest quantity of lychees [1,2]. China possesses the most extensive collection of high-quality lychee germplasm resources globally due to its long history of cultivation. At the same time, people have always liked lychee because of its high nutritional value, unusual taste, and bright color. It is one of the most popular tropical and subtropical fruits internationally [3,4,5]. As lychee farming has expanded, so has the incidence of diseases and pests. Lychee’s downy blight, which is caused by the pathogen Peronophytora lychee, plays a crucial role as a significant disease in the production of lychee fruit. This disease not only happens when the plant is growing, putting fruits, flower spikes, and tender leaves at risk, but it also happens when the fruit is being stored, causing the fruit to rot. This greatly affects the quality, yield, and sale of fresh lychee fruit [6]. The best way to avoid and control lychee’s downy blight in production is through chemical control, as it works effectively and rapidly. This is because there are not enough types of lychee with good agricultural traits, high output, and disease protection [7].

**Figure 1:**
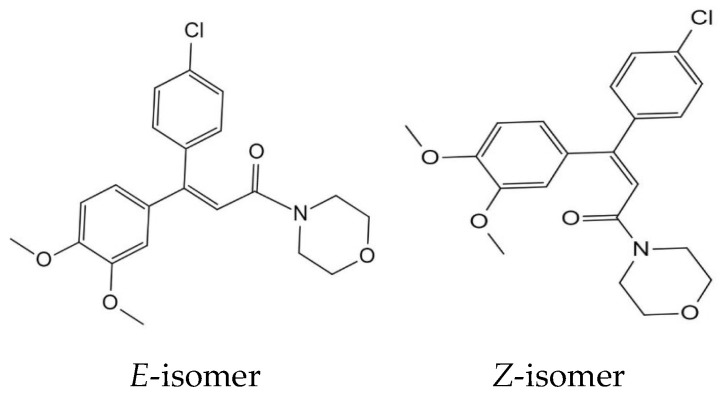
The structure of dimethomorph.

The efficacy of carboxylic acid amides (CAAs) as fungicides has garnered significant interest owing to their potent antibacterial properties, robust internal absorption conductivity, and selective impact on egg bacteria [7]. Dimethomorph is a fungicide in the CAA group (Figure 1). The NOAEL of dimethomorph was 100 mg/kg bw per day on the basis of body weight gain decreases at the highest dose. On the basis of the absence of carcinogenicity and genotoxicity, the potential hazards of dimethomorph for foetuses, infants, and children were deemed adequate. Dimethomorph is stable under hydrolysis and photolysis and is moderately persistent in soil, with field half-lives of 10–61 days. In rotational crops, dimethomorph can be taken up by the roots and dimethomorph residues may occur in early harvest crops (e.g., spinach) planted within 44 days of the last application [8]. Fungicides in this group have both protective and healing benefits and are used a lot to stop lychee’s downy blight and keep it from spreading. As of 31 May 2023, China has 78 lychee fungicides, 3 of which included dimethomorph, accounting for 4%. Health concerns are developing among consumers, and environmental consequences are associated with the presence of pesticide residues. Hence, it is imperative to undertake a study on the attributes of pesticide residuals and the evaluation of dietary hazards in lychee.

Based on our current understanding, several studies have investigated the detection of dimethomorph residue in various agricultural products such as cucumber, grape, and ginseng [9,10,11]. Dimethomorph had half-lives of 1.5–4.7 days in cucumbers and 17.7–33.6 days in soil. Final residues ranged between 0.006 and 0.632 mg/kg and between 0.108 and 4.866 mg/kg [9]. According to a study, the half-lives of dimethomorph in grapes were found to be between 7.3 and 14.8 days, with final residues measuring less than 0.5 mg/kg [10]. The study determined that the degradation half-life of dimethomorph in various parts of ginseng, including roots, stems, leaves, and soil, ranged from 9.13 to 16.35 days. According to a study, the presence of dimethomorph in ginseng roots, stems, leaves, and soil was found to be negligible, with residual amounts ranging from undetectable to 0.025, 0.023 to 0.139, 0.122 to 0.618 mg/kg, and 0.008 to 0.074 mg/kg, respectively [11]. GC-ECD and HPLC-MS/MS were utilized for the aforementioned procedures. The primary pre-treatment techniques employed were liquid–liquid distribution extraction, packed column, and solid-phase extraction (SPE). The liquid–liquid distribution extraction technique uses a substantial quantity of organic solvents, which is susceptible to emulsification, leading to a reduction in recovery efficiency. The packed column technique is a time-consuming and labor-intensive process that requires vast amounts of organic solvents. SPE also tends to be a lengthy process. The precision and dependability of pesticide residue tests are directly related to the quality of the sample pretreatment procedures used. Food pretreatment techniques include solid-phase microextraction (SPME) [12,13], SPE [14,15], MSPD [16], and QuEChERS (quick, easy, cheap, effective, rugged, and safe) [17,18,19,20,21]. The QuEChERS method integrates a small amount of adsorbent material into the extraction solution, which effectively seizes impurities. This technique is characterized by its simplicity, stability, environmental friendliness, cost-effectiveness, and efficacy [22]. The predominant approach employed for identifying pesticide residues in diverse matrices is the utilization of HPLC-MS/MS techniques [23], HPLC [24], gas chromatography (GC) [25], and gas chromatography–mass spectrometry (GC-MS) [26]. Dimethomorph’s detection limits were determined to be 0.01 mg/kg in grapes through the use of GC-ECD. Similarly, in cucumber and ginseng, the detection limits were found to be 0.001–0.006 mg/kg through the utilization of HPLC-MS/MS. The utilization of HPLC-MS/MS has been recognized as a proficient technique for the detection of pesticide residues due to its superior resolution, selectivity, and potency. This approach can detect a wide range of pesticides in various food products, making it an effective means of analysis. Typically, QuEChERS is employed in conjunction with HPLC-MS/MS for this purpose. To date, there has been a lack of research conducted on the evaluation of dissipation patterns and terminal residues of dimethomorph in lychee crops grown in open-field settings. Therefore, it holds significant theoretical and practical importance to elucidate the residual properties of dimethomorph in the primary lychee production regions.

The maximum residue limit (MRL) of dimethomorph on lychee has yet to be established by the Codex Alimentarius Commission (CAC), as well as by the United States and China. Regulatory limits known as MRLs are implemented to manage the concentrations of pesticide residues on food commodities. In cases where pesticide residue levels exceed the predetermined MRL thresholds, it is anticipated that they may present a possible hazard to both humans and animals. Additionally, conducting a dietary risk evaluation of pesticide residues present in food items can establish a scholarly foundation for ensuring secure production, offering consumption recommendations, overseeing safety, and determining MRL. Nonetheless, the number of investigations conducted on the evaluation of dietary hazards associated with pesticide residues in Chinese lychee remains limited. Hence, a methodology was developed and verified for the identification of dimethomorph on lychee fruit through the utilization of QuEChERS in conjunction with HPLC-MS/MS. The developed approach was utilized to identify pesticides on lychee samples, and the findings were used to assess the risk levels related to the dietary consumption of Chinese consumers.

The official regulation of the MRL of dimethomorph, a potent fungicide, in lychee is absent in China. Additionally, attempts to quantify the residue levels of this pesticide in lychee have been notably absent. The present study aims to address the aforementioned gaps in knowledge by pursuing multiple objectives. The first objective of this study is to refine an HPLC-MS/MS-based method for detecting dimethomorph in lychee samples, thereby establishing a reliable measurement technique. Second, it examines dimethomorph’s dissipation kinetics, terminal residues, and distribution in lychee and pulp under field settings. Finally, using terminal residue analysis data, it will analyze dimethomorph safety in lychee and calculate risk quotient (RQ) values. 

## 2. Results and Discussion

### 2.1. Analytical Method Verification

**Figure 2:**
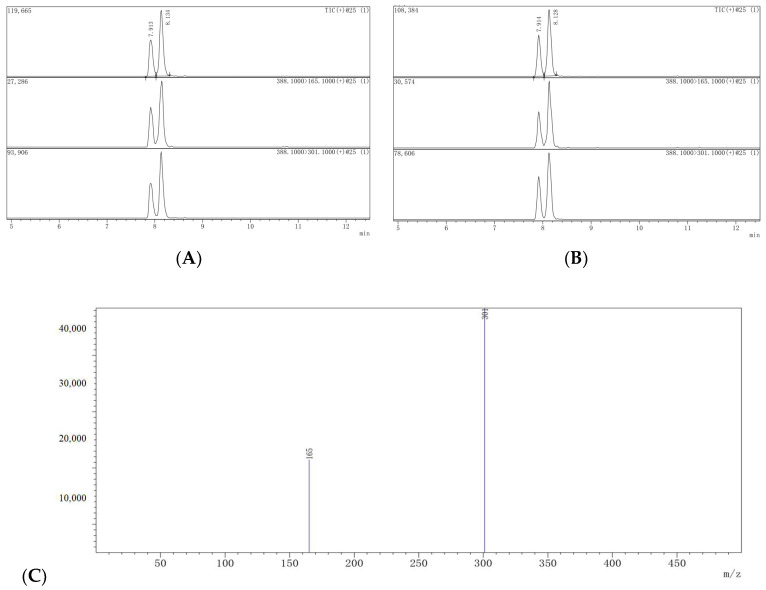
Chromatograms and MS spectra of dimethomorph standard and spiked sample (lateral axis is time, and longitudinal axis is response intensity). (**A**) Dimethomorph standard (0.05 mg/kg); (**B**) spiked lychee sample (0.05 mg/kg); (**C**) MS spectra of dimethomorph standard (0.05 mg/kg).

The analytical method was validated through the assessment of several parameters, including linearity, recovery, precision, and accuracy. The method’s accuracy and precision were evaluated through the determination of recoveries. The present study evaluated the linearity of whole lychee and pulp using matrix-matched samples within the concentration range of 0.001–0.5 mg/L. The coefficient of correlation r^2^ was equal to 1.000. Table 1 displays the accuracy and precision data that were derived from recovery studies consisting of five replicate samples (*n* = 5) at three distinct dimethomorph levels. The results indicate that the proposed method for pesticide residue analysis meets the standard validation requirements. The mean recoveries for all concentration levels were found to be satisfactory, with a range of 77–90% and RSDs of 2–10% in the whole lychee and 89–92% with RSDs of 5–6% in the pulp. The LOQs for whole lychee and pulp were 0.001 mg/kg (Table 1). The findings suggest that the method falls within the acceptable range, exhibiting commendable accuracy and repeatability. Figure 2 displays the HPLC-MS/MS chromatograms for both blank and spiked samples, serving as representative examples.

### 2.2. Dissipation of Dimethomorphs in Lychee

**Figure 3:**
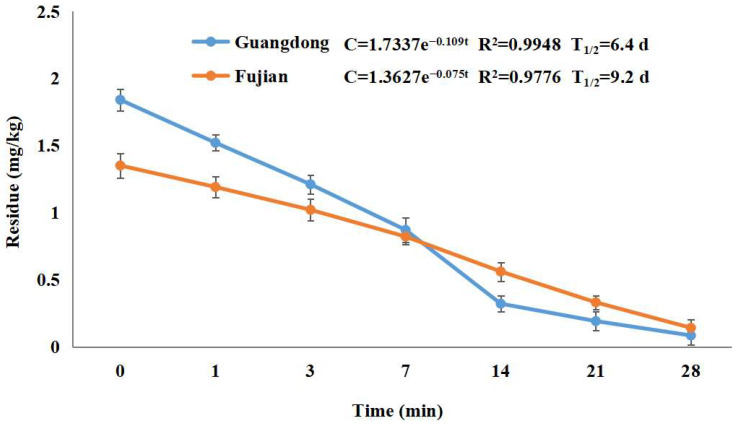
The dissipation pattern of dimethomorph in lychee (lateral axis is collected sample time, and longitudinal axis is residues).

Figure 3 displays the dissipation pattern of dimethomorph in lychee under open-field conditions. The findings of the study revealed that the lychee fruit cultivated in the Guangdong region exhibited a greater concentration of dimethomorph in comparison to those grown in the Fujian region. Specifically, the initial deposit of dimethomorph was recorded as 1.84 mg/kg in the Guangdong region and 1.35 mg/kg in the Fujian region. The concentration of dimethomorph in the lychee treatment in Guangdong exhibited a significant decline from day 0 to day 7 post-application, ultimately reaching a level of 0.87 mg/kg. This decrease was accompanied by a substantial loss of 52.7%. After 28 days of application, the degradation of dimethomorph exhibited a rapid decline, with a reduction to levels below 0.083 mg/kg and a corresponding loss of 95.5%. In the Fujian region, the concentration of dimethomorph residues was found to be 0.14 mg/kg after 28 days of application, with a dissipation dynamic of 89.6%, according to the employed methodology. The experimental sites exhibited distinct climatic conditions, with Guangdong and Fujian experiencing average temperatures ranging from 20 to 40 °C and from 21 to 36 °C, respectively. The average precipitation during the experiment was 514 mm and 189 mm for Guangdong and Fujian, respectively. The study determined the degradation half-life (t_1/2_) of dimethomorph in lychee through a regression equation. The calculated t_1/2_ values were 6.4 and 9.2 days in Guangdong and Fujian, respectively. These values were comparable to those found in cucumbers (7.3–14.8 d) [9], but slower than grapes (1.5–4.7 d) [10] and faster than ginseng (9.13–16.35 d) [11]. The dissipation of dimethomorph residues can be attributed to various physical and chemical factors, including but not limited to temperature, precipitation, sunlight, pH, and planting spacing. Furthermore, it has been reported by certain researchers that the decline of residues may also be influenced by the growth dilution factor [27].

### 2.3. Lychee Dimethomorph Terminal Residues

Table 2 presents a summary of the terminal residue of dimethomorph in both whole lychee and pulp samples that were collected from the treated plots. The investigation focused on the terminal residues of dimethomorph in both whole lychee and pulp, specifically at 5, 7, and 10 days following the final application. Upon application of dimethomorph at the recommended dosage of 266.6 mg/kg, the resulting residues in whole lychee were found to range from 0.19 to 1.21, 0.15 to 0.96, and 0.09 to 0.75 mg/kg, while the residues in the pulp were found to range from 0.019 to 0.13, 0.012 to 0.092, and 0.009 to 0.054 mg/kg, based on pre-harvest intervals of 5, 7, and 10 days. The trend of decreasing residues with increasing PHIs was readily observable in the concentration data. Dimethomorph was administered at 1.5 times the recommended dosage (399.9 mg/kg) for 5, 7, and 10 days. The resulting concentrations of dimethomorph were observed to be within the ranges of 0.39–1.99, 0.29–1.56, and 0.19–1.23 mg/kg in the entirety of the lychee fruit, and within the ranges of 0.054–0.18, 0.038–0.12, and 0.018–0.085 mg/kg in the pulp, respectively. The findings indicated a positive correlation between residues and the frequency and quantity of application. The concentration of dimethomorph residues in the pulp was found to be lower compared to that in the whole lychee. This observation suggests that the majority of the residues were likely to be localized in the peel. As a result of the application of dimethomorph WG onto the peel, the pulp was not subjected to direct exposure to the pesticide. In contrast, dimethomorph is a fungicide that is soluble in fat, as evidenced by its K_ow_ logP range of 2.63–2.73 at a temperature of 20 °C, according to *The E-pesticide Manual*, Version 3.0. This property makes it highly prone to absorption by the peel. Consequently, the levels of residues that infiltrated the pulp were found to be minimal. It is reported that the residue distribution of most pesticides conforms to the fact that the residue in the whole fruit is higher than that in the pulp, but the residue of carbendazim, methomyl, and thiabendazole in the pulp is higher than that in the whole fruit. The distribution of pesticides in fruits is influenced by various factors such as pesticide properties, environmental conditions, fruit properties, application techniques, and agronomic measures [28]. The pesticides in pulp mainly come from the transportation of branches and leaves and easily accumulate in the flesh after entering the fruit through the stem. The surface of the peel is usually coated with a layer of wax, which has strong hydrophobicity and can prevent hydrophilic pollutants from entering the interior of the fruit, which is the main cause of low pesticide residues in pulp [29]. Based on the findings, it can be inferred that the levels of dimethomorph residues in lychee fruit exhibit an upward trend with increasing application dosage. However, the variations in residue levels across the six regions were not statistically significant. The timing of the harvest season was a significant factor in determining the terminal residues. Following a 7-day application period, the concentrations of dimethomorph residues in all of the lychee samples were found to be below 1.56 mg/kg when applied at both the recommended dosage and 1.5 times the recommended dosage. The median residue concentration was determined to be 0.72 mg/kg. Based on this, it is recommended that the maximum limit of dimethomorph on lychee is 5 mg/kg. To prevent and control lychee downy blight, 50% dimethomorph WG is used with a maximum dosage of 399.9 mg/kg, a maximum of three applications, and a PHI of 7 days.

### 2.4. Risk Evaluation

The investigation of risk assessment for dimethomorph in lychee has been conducted, taking into account the customary consumption of food (fruit) in China [30]. The process of risk assessment involves a crucial step known as dietary exposure assessment, which encompasses both acute and chronic exposure assessment [31]. The assessment of dimethomorph exposure via consumption of lychee was conducted utilizing the RQ approach. The JMPR report was utilized to derive ARfD (0.6 mg/kg, bw) and ADI values (0.2 mg/kg, bw) to conduct a risk assessment on dimethomorph residues present in pulp samples. This research examined the hazards associated with the consumption of lychee contaminated with dimethomorph, both in the short term and long term. The results indicated that the values of the acute reference dose percentage (ARfD%) and acceptable daily intake percentage (ADI%) ranged from 0.0039% to 0.0994% and from 0.0015% to 0.1822%, respectively. These findings suggest that the risks associated with exposure to dimethomorph through lychee consumption are within acceptable limits both in terms of acute and chronic toxicity. According to the data presented in Table 3, it was observed that dimethomorph exhibited a greater acute reference dose percentage (ARfD%) and acceptable daily intake percentage (ADI%) for children aged 2–4 years. Additionally, the risk quotient was found to increase with an increase in the frequency and concentration of application, while it decreased with a longer pre-harvest interval (PHI).

## 3. Material and Methods

### 3.1. Chemicals and Reagents

Dimethomorph (98.7%) standard was purchased from Dr. Ehrenstorfer GmbH (Augsburg, Germany). A water-dispersible granule (WG) containing 50% dimethomorph was obtained from Zhonggang Wanxiang Agricultural Co., Ltd. (Zhengzhou, China). Formic acid of chromatographic purity was acquired from Fluka (Newport News, VA, USA). Acetonitrile and methanol of HPLC grade were supplied by Thermo Fisher Co., Ltd. (Waltham, MA, USA) Analytical-grade sodium chloride and activated anhydrous sodium sulfate were obtained from Sigma-Aldrich (Steinheim, Germany). The graphitized carbon black adsorbent (GCB, 120–400 mesh) was sourced from ANPEL Laboratory Technologies Inc. (Shanghai, China).

Acetonitrile was used to make standard pesticide stock solutions with a concentration of 1000 mg L^−1^, which were then kept at −20 °C. Acetonitrile was used to dilute the stock solution to generate the standard working solutions required for fortification and calibration, which were made fresh as needed. The solvent calibration and matrix-matched solutions were made by diluting the stock solutions with acetonitrile and blank extracts from all matrices, respectively. Before use, all standard solutions were kept at 4 °C.

### 3.2. Field Research and Sample Preparation

**Figure 4:**
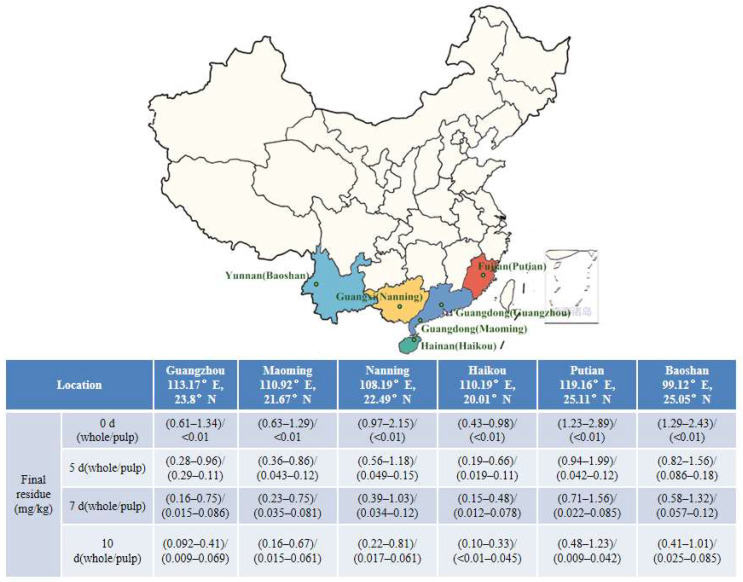
Geographical locations and final residues of the sampling sites of lychee samples in China.

In 2018, field examinations were carried out in six areas (Guangzhou and Maoming in Guangdong Province, Nanning in Guangxi Province, Baoshan in Yunnan Province, Putian in Fujian Province, and Haikou in Hainan Province), including residue dynamic investigations and terminal residue evaluations (Figure 4). These were carried out in accordance with the “Guideline on Pesticide Residue Trials” issued by the Ministry of Agriculture of the People’s Republic of China (NY/T788-2018), as well as the pesticide labels’ recommendations.

Applications were made for the terminal residue assessment at the supervised field trial at the approved dose of 266.6 mg/kg, as well as a higher dosage of 399.9 mg/kg, which is 1.5 times the recommended dosage. At both the lower and higher doses, a 50% dimethomorph WG was sprayed twice and three times, with a 7-day gap between each treatment. Each plot had two lychee trees, and each treatment had three replication plots and one control plot. Following the final spraying, representative lychee samples (2 kg) were collected at preharvest intervals (PHIs) of 5, 7, and 10 days from different sites within each plot. The whole lychee fruit and its pulp were gathered for the terminal residue studies. After that, the lychee samples were quartered and homogenized for examination.

A 50% dimethomorph WG solution in water was sprayed over the lychee plots at a concentration of 399.9 mg/kg when the lychee was roughly 50% the size of a mature fruit to evaluate dimethomorph dissipation. Simultaneously, a plot of the same size but without dimethomorph treatment was compared. At 2 h, 1, 3, 7, 14, 21, and 28 days following treatment, representative lychee samples were randomly obtained from each plot for dissipation assessment. All samples were stored at −20 °C for later examination.

Each sample included at least 2 kg of lychee. Prior to processing, all extraneous elements, such as withered leaves and surface debris, were removed. Without washing or peeling, the samples were immediately homogenized in a food processor after collection. Homogenized samples were frozen at −20 °C in sealed polyethylene vials. The frozen samples were immediately sent to the laboratory in a sealed container packed with sufficient ice and maintained in a frozen state until analysis. All samples were evaluated within one month after being homogenized.

### 3.3. Cleanup and Extraction

A 5 g sample of either whole lychee fruit or pulp was put in a 50 mL centrifuge tube for extraction. An 80% acetonitrile aqueous solution (10 mL) was then added, and the mixture was forcefully agitated for 1 min using a vortex mixer set to 5204× *g*. After 3 g of NaCl was added, the samples were placed in a 40 °C water bath for a 30 min extraction. They were then violently mixed for 1 min before being centrifuged for 5 min at 3800 rpm. Following that, a 2 mL portion of the supernatant was transferred to a 5 mL centrifuge tube containing 150 mg anhydrous MgSO_4_ and 50 mg GCB for cleaning. This was followed by another minute of shaking and 5 min of centrifugation at 8000 rpm. A 0.22 micrometer nylon syringe filter was used to filter the sample’s supernatant before transferring it to an autosampler vial for HPLC-MS/MS analysis.

### 3.4. Instrumentation and HPLC Analytical Specifications

The separation of the target compounds was carried out utilizing an Agilent Extend C18 column (4.6 mm × 150 cm, 2.5 μm, Agilent, Santa Clara, CA, USA) in conjunction with a Shimadzu LC-20A HPLC system, with the column oven being maintained at a temperature of 35 °C. The separation technique employed gradient elution, whereby the mobile phases A (aqueous solution) and B (MeCN) were utilized. Throughout the study, the flow rate was kept constant at 0.3 mL/min. The gradient program was as follows: 80% A–25% A for the first 3 min, 25% A–5% A for the next 3–14 min, 5% A for the next 14–14.5 min, and 80% A for the last 14.5–19 min. An autosampler was used to carry out the injection (1 μL).

For the measurement of target analytes, a triple quadrupole mass spectrometer (Shimadzu 8045; Shimadzu; Kyoto, Japan) equipped with positive ion mode electrospray ionization (ESI^+^) was used. The temperature of the interface was set to 300 °C, the desolvation line to 526 °C, and the heating block to 400 °C. Nitrogen was employed as a nebulizer as well as a crash gas. MRM (multi-reactive ion monitoring) was chosen for target analyte analysis. By immediately injecting a 100 mg/L standard solution of each target pesticide into the instrument’s ion source, the ideal precursor ions, product ions, collision energies, and other instrument parameters for dimethomorph were 388.1, 301.1/165.1, and −21/−33, respectively.

### 3.5. Methodology Validation

The proposed techniques’ accuracy and reliability were assessed using recovery rate, linear equation, and limit of quantification (LOQ) values. The linearity of the developed standards was in the 0.001–0.5 mg L^−1^ range. Blank food samples were spiked with dimethomorph at dosages of 0.001, 0.01, or 0.1 mg/kg, with five replicates per concentration. This analysis’s relative standard deviations (RSDs) were utilized to evaluate technique precision. According to the SANTE/11813/2017 criteria [32], the LOQ for dimethomorph was established as the lowest spiking level observable in the selected matrix.

### 3.6. Statistical Analysis

By tracking the residue concentration versus time, the dissipation dynamics of dimethomorph in lychee were determined. The best-fit curve equations were derived using the greatest squares of correlation coefficients discovered. The equation C_t_ = C_0_^ekt^, where C_t_ is the pesticide residue concentration at time t, C_0_ is the starting concentration after application, and k is the constant for dissipation degradation rate, provided pictorial validation of the first-order kinetics. The dissipation half-lives (t_1/2_) were computed as ln2/k. All results are shown as the mean, standard deviation (SD) of five replicates.

Dietary exposure estimates and risk evaluations were performed using the RQ approach to ensure the safe usage of dimethomorph. An RQ number of more than one indicated that the pesticide danger to people was unacceptable, whilst a value less than one showed that the pesticide risk to humans was minor. The acceptable daily intake (ADI, mg/kg/d) and the median pesticide residue levels discovered on lychee were used to compute the chronic dietary exposure assessment, which takes into account risk over a lifetime. The chronic risk quotient, ADI%, was computed using the following formula [25]:

ADI% = (STMR × FI)/(ADI × bw) × 100%,

STMR (mg/kg) refers to the supervised trials’ median residues; FI (kg/day) is the dietary reference intake for a certain kind of food, which is used to estimate the nutritional intakes of healthy Chinese adults; and bw (kg) is the mean body weight. According to the China Health and Nutrition Survey, the average adult Chinese body weight is 63 kg.

The HR and the acute reference dose (ARfD, mg/kg/d) were used to generate the acute dietary exposure assessment, which predicts the consumer health risk of pesticide ingestion via food in the short term. The acute risk quotient, ARfD%, was calculated using the following formula:

ARfD% = (HR × FI)/(ARfD × bw) × 100%,

HR (mg/kg) in this equation refers to the maximum recorded pesticide residue levels identified on lychee (with a value of 0 for undetected samples). Dimethomorph has an ARfD of 0.6 mg/kg/d. 

## 4. Conclusions

The quantification of dimethomorph residue present in whole lychee and pulp can be accomplished through the utilization of the QuEChERS method in conjunction with HPLC-MS/MS. Dimethomorph was subjected to an assessment to determine its dissipation, terminal residue, distribution, and risk. The objective of this evaluation was to ensure the judicious and secure application of dimethomorph. The findings indicate that dimethomorph exhibited a swift dissipation dynamic, with a half-life ranging from 6.4 to 9.2 days in lychee, conforming to the principles of first-order kinetics. The terminal residues in the whole lychee were much more than in the pulp, which indicates that dimethomorph residues were mainly concentrated in the peel. MRL was recommended according to the final residue results of dimethomorph in the whole lychee. According to the acute and chronic risk quotient value, the potential harm to humans from dimethomorph at the recommended dosage was deemed negligible, with a value lower than one. This study has the potential to serve as a valuable resource for the appropriate application of dimethomorph in lychee cultivation. The findings may aid in the establishment of a logical MRL for dimethomorph in lychee.

## Figures and Tables

**Table 1:** Performance characteristics of the method for dimethomorph in the whole lychee and pulp.

Matrix	Fortified Level (mg/kg)	Average Recovery (%, *n* = 5)	RSDa (%)	RSDr (%)	Correlation Coefficient	LOQ (mg/kg)
lychee	0.001	83	7	10	1	0.001
	0.01	90	5	8		
	0.1	77	3	6		
pulp	0.001	89	4	11	0.9995	0.001
	0.01	89	5	10		
	0.1	92	3	8		

Note: RSDa is intra-day precisions (*n* = 5), RSDr is inter-day precisions (*n* = 15).

**Table 2:** Terminal residues of dimethomorph during different PHI in whole lychee and pulp.

Dosage (mg/kg)	Spray Times	Mean, Median, and HR Residues in Whole Lychee (mg/kg)			Mean, Median, and HR Residues in Pulp (mg/kg)		
		PHI = 5	PHI = 7	PHI = 10	PHI = 5	PHI = 7	PHI = 10
266.6	2	0.57/0.49/1.03	0.42/0.37/0.85	0.28/0.22/0.62	0.054/0.051/0.11	0.034/0.035/0.075	0.019/0.020/0.042
	3	0.68/0.61/1.21	0.52/0.46/0.93	0.39/0.35/0.75	0.067/0.062/0.13	0.049/0.047/0.092	0.028/0.041/0.054
399.9	2	0.92/0.86/1.77	0.76/0.75/1.25	0.56/0.59/0.96	0.096/0.096/0.16	0.068/0.062/0.11	0.044/0.044/0.064
	3	1.10/1.00/1.99	0.89/0.90/1.56	0.67/0.68/1.23	0.12/0.11/0.18	0.083/0.077/0.12	0.049/0.050/0.085

**Table 3:** The risk evaluation of dimethomorph in lychee.

**Application Dosage (mg/kg)**	**Spray Times**	**PHI** **(Days)**	**ARfD** **(mg/kg/d)**	**HR** **(mg/kg)**	**%ARfD**					
					**2~4 Male**	**2~4 Female**	**18~30 Male**	**18~30 Female**	**60~70 Male**	**60~70 Female**
266.6	2	5	0.6	0.11	0.0568	0.0607	0.0127	0.0184	0.0101	0.0117
		7		0.075	0.0387	0.0414	0.0086	0.0126	0.0069	0.008
		10		0.042	0.0217	0.0232	0.0048	0.007	0.0039	0.0045
	3	5		0.13	0.0671	0.0718	0.015	0.0218	0.0119	0.0139
		7		0.092	0.0475	0.0508	0.0106	0.0154	0.0085	0.0098
		10		0.054	0.0279	0.0298	0.0062	0.0091	0.005	0.0058
399.9	2	5		0.16	0.0826	0.0884	0.0184	0.0268	0.0147	0.0171
		7		0.11	0.0568	0.0607	0.0127	0.0184	0.0101	0.0117
		10		0.064	0.0331	0.0353	0.0074	0.0107	0.0059	0.0068
	3	5		0.18	0.093	0.0994	0.0207	0.0302	0.0165	0.0192
		7		0.12	0.062	0.0663	0.0138	0.0201	0.011	0.0128
		10		0.085	0.0439	0.0469	0.0098	0.0142	0.0078	0.0091
**Application dosage** **(mg/kg)**	**Spray times**	**PHI** **(days)**	**ADI** **(mg/kg/d)**	**MMR** **(mg/kg)**	**%ARfD**					
					**2~4 male**	**2~4 female**	**18~30 male**	**18~30 female**	**60~70 male**	**60~70 female**
266.6	2	5	0.2	0.051	0.0790	0.0845	0.0176	0.0256	0.0141	0.0163
		7		0.035	0.0542	0.0580	0.0121	0.0176	0.0096	0.0112
		10		0.020	0.0310	0.0331	0.0069	0.0101	0.0055	0.0064
	3	5		0.062	0.0961	0.1027	0.0214	0.0312	0.0171	0.0199
		7		0.047	0.0728	0.0779	0.0162	0.0236	0.0130	0.0151
		10		0.041	0.0635	0.0679	0.0142	0.0206	0.0113	0.0131
399.9	2	5		0.096	0.1488	0.1590	0.0332	0.0483	0.0265	0.0308
		7		0.062	0.0961	0.1027	0.0214	0.0312	0.0171	0.0199
		10		0.044	0.0682	0.0729	0.0152	0.0221	0.0121	0.0141
	2	5		0.110	0.1705	0.1822	0.0380	0.0553	0.0303	0.0352
		7		0.077	0.1193	0.1276	0.0266	0.0387	0.0212	0.0247
		10		0.050	0.0775	0.0828	0.0173	0.0251	0.0138	0.0160

## Data Availability

Data are contained within the article.
